# Induction of human airway epithelial to mesenchymal transition upon rhinovirus infection

**DOI:** 10.1186/1710-1492-10-S1-A53

**Published:** 2014-03-03

**Authors:** Danielle Minor, Suzanne Traves, David Proud, Richard Leigh

**Affiliations:** 1 Snyder Institute for Chronic Diseases, University of Calgary, Calgary, Alberta, T2N 4N1, Canada

## Background

Structural changes of the airway, collectively referred to as airway remodeling are believed to be the underlying cause of the airway hyperresponsiveness that is characteristic of asthma. Airway remodeling is characterized by thickening of the subepithelial membrane, goblet cell hyperplasia, angiogenesis, increased smooth muscle mass, and epithelial fragility. Thickening of the subepithelial membrane, due to increased deposition of matrix proteins by fibroblasts/myofibroblasts, has been observed in children even prior to the formal diagnosis of asthma. Recent data have shown that young children who experience episodes of human rhinovirus (HRV)-induced wheezing in early childhood are at increased risk of subsequently developing asthma. The primary site of infection of HRV is the airway epithelium. Recent evidence suggests that the molecular reprogramming of epithelial cells through a process called epithelial to mesenchymal transition (EMT) may contribute to increases in fibroblast/myofibroblast in the asthmatic airway. Therefore, we hypothesize that HRV infection plays a role in early airway remodeling by triggering EMT to produce fibroblasts/myofibroblasts that cause thickening of the subepithelial membrane by depositing matrix proteins.

## Methods

The BEAS-2B human bronchial epithelial cell line was grown in 6-well plates in bronchial epithelial growth medium (BEGM). Prior to experiments, cells were grown for 24 h in BEGM from which hydrocortisone was removed. Cells were exposed to medium (control), or purified HRV-16 alone or in the presence of various growth factors for 120 hours with media and growth factor replacement at 48 and 96 hours. Cell lysates were collected and then analyzed by western blot for protein expression of epithelial and mesenchymal markers.

## Results

BEAS-2B cells that were infected with HRV in the presence of epidermal growth factor (EGF) showed decreased expression of the epithelial marker E-cadherin, and increased expression of the mesenchymal marker vimentin.

## Conclusions

HRV infection, particularly in combination with EGF causes changes characteristic of EMT. I will confirm and extend these observations by looking at other epithelial and mesenchymal markers and looking for phenotypic changes. Future studies will examine the mechanisms underlying HRV induced EMT using siRNA and pharmacologic approaches.

